# Identification and Characterization of the Actin-Binding Motif of Phostensin

**DOI:** 10.3390/ijms131215967

**Published:** 2012-11-28

**Authors:** Tzu-Fan Wang, Ning-Sheng Lai, Kuang-Yung Huang, Hsien-Lu Huang, Ming-Chi Lu, Yu-Shan Lin, Chun-Yu Chen, Su-Qin Liu, Ta-Hsien Lin, Hsien-Bin Huang

**Affiliations:** 1 Department of Life Science and Institute of Molecular Biology, National Chung Cheng University, Chia-Yi 62102, Taiwan; E-Mails: catalpa0905@gmail.com (T.-F.W.); lambdaphagecarol@yahoo.com.tw (Y.-S.L.); biogoldfish@mail2000.com.tw (C.-Y.C.); 2 College of Medicine, Tzu-Chi University, Hualien 97004, Taiwan; E-Mails: q12015@tzuchi.com.tw (N.-S.L.); hky0919@yahoo.com.tw (K.-Y.H.); dm252940@tzuchi.com.tw (M.-C.L.); 3 Section of Allergy, Immunology and Rheumatology, Department of Medicine, DaLin Tzu Chi Buddhist Hospital, Chia-Yi 62247, Taiwan; E-Mail: t480710@yahoo.com.tw; 4 Department of Nutrition and Health Science, Fooyin University, Kaohsiung 83102, Taiwan; E-Mail: estrus@mail2000.com.tw; 5 Institute of Biochemistry and Molecular Biology, National Yang-Ming University, Taipei 11221, Taiwan; 6 Department of Medical Research & Education, Taipei Veterans General Hospital, Taipei 11217, Taiwan

**Keywords:** phostensin, actin filament, *KIAA1949*, protein phosphatase 1

## Abstract

Phostensin, a protein phosphatase 1 F-actin cytoskeleton-targeting subunit encoded by *KIAA1949*, consists of 165 amino acids and caps the pointed ends of actin filaments. Sequence alignment analyses suggest that the *C*-terminal region of phostensin, spanning residues 129 to 155, contains a consensus actin-binding motif. Here, we have verified the existence of an actin-binding motif in the *C*-terminal domain of phostensin using colocalization, F-actin co-sedimentation and single filament binding assays. Our data indicate that the *N*-terminal region of phostensin (1–129) cannot bind to actin filaments and cannot retard the pointed end elongation of gelsolin-actin seeds. Furthermore, the *C*-terminal region of phostensin (125–165) multiply bind to the sides of actin filaments and lacks the ability to block the pointed end elongation, suggesting that the actin-binding motif is located in the *C*-terminal region of the phostensin. Further analyses indicate that phostensin binding to the pointed end of actin filament requires *N*-terminal residues 35 to 51. These results suggest that phostensin might fold into a rigid structure, allowing the *N*-terminus to sterically hinder the binding of *C*-terminus to the sides of actin filament, thus rendering phostensin binding to the pointed ends of actin filaments.

## 1. Introduction

Phostensin, a protein phosphatase 1 F-actin cytoskeleton targeting subunit, encoded by *KIAA1949*, consists of 165 amino acids with a consensus protein phosphatase 1 (PP1)-binding motif located at residues 91–94 (K_91_ISF_94_) [1]. The location of the phostensin gene is between the *HLA*-*C* and *HLA*-*E* gene clusters on human chromosome 6 [2,3]. Phostensin mRNA has been found in peripheral leukocytes, the thymus and the spleen [1,4]. Phostensin is a ubiquitous protein in mature human leukocytes and leukemia cell lines [5]. It is mainly concentrated at the cell periphery and colocalizes with actin filaments and protein phosphatase 1 (PP1). Phostensin binds to the pointed ends of actin filaments, but not to actin monomers, the sides of filaments or the barbed ends of filaments [1,5,6]. Results from a previous study suggest that phostensin retards the elongation and depolymerization rates of gelsolin-actin seeds, directly associates with pointed actin filament ends and reduces the rate of G-actin addition at these pointed ends [6].

Actin is an abundant protein in eukaryotic cells [7–9]. Monomeric actin, called G-actin, polymerizes into a double helical filament called F-actin. Since F-actin is a polar molecule, the two ends of the actin filaments have different growing rates under physiological conditions, forming distinct structures. Several types of actin-binding proteins (ABPs) have been found to bind to different sites on actin (including monomeric G-actin), actin filament caps and filament sides. Such actin-binding proteins can modulate the actin dynamics that play essential roles in a variety of cellular functions. ABPs contain a consensus actin-binding motif [10], L(X)_7_(D/E)(X)_6_L(X)_2_(E/D) L(X)_3_L(X)_2_(K/R), which is found in F-actin barbed end-capping proteins, actin filament severing proteins and G-actin-binding proteins [10–13]. However, there are no reports that indicate whether this motif is also present in the F-actin pointed end-capping protein. In the current study, we find that phostensin, an F-actin pointed end-capping protein, also contains a consensus actin-binding motif, located in the *C*-terminal domain. The F-actin-binding properties of this consensus motif were further analyzed, demonstrating that this motif is required for phostensin binding to actin filaments. However, phostensin mutants, which lack the *N*-terminal domain, bind to the sides of actin filaments, suggesting that the *N*-terminal region of phostensin is responsible for capping the pointed ends of actin filaments.

## 2. Results and Discussion

### 2.1. Sequence Analyses

**Figure 1:**
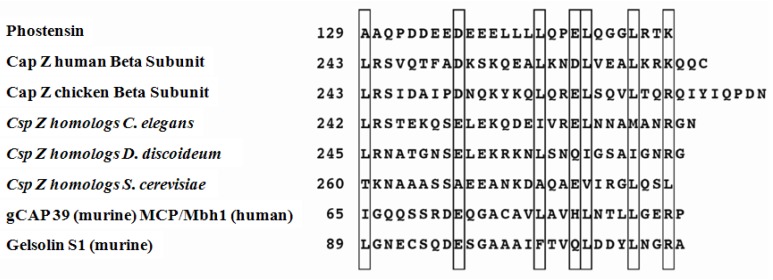
Sequence comparisons between the *C*-terminal domain of phostensin and other F-actin capping proteins. The consensus sequence for the actin capping motif, L(X)_7_(D/E)(X)_6_L(X)_2_(E/D)L(X)_3_L(X)_2_(K/R), was defined by Barron-Casella *et al.*[10]. The numbers of the first residues of the aligned sequences are given. Boxes indicate identical or conservatively substituted residues among the aligned F-actin capping proteins.

Fluorescence microscopy and immunostaining revealed that phostensin colocalized with the actin cytoskeleton at the cell periphery in MDCK epithelial cells and in human peripheral mononuclear cells (PBMCs) [1,5]. In addition, actin could be immunoprecipitated from PBMC extracts using an anti-phostensin monoclonal antibody. These results demonstrated that phostensin is an actin-binding protein [5]. Sequence analyses suggest that the *C*-terminal domain, spanning phostensin residues 129 to 155, contains an actin-binding motif with conserved amino acids in a leucine-rich array (Figure 1), which is often found in actin-binding proteins [10]. This result further supports the observation that phostensin is an actin-binding protein [5].

### 2.2. Cellular Colocalization and Actin Co-Sedimentation Assays

**Figure 2:**
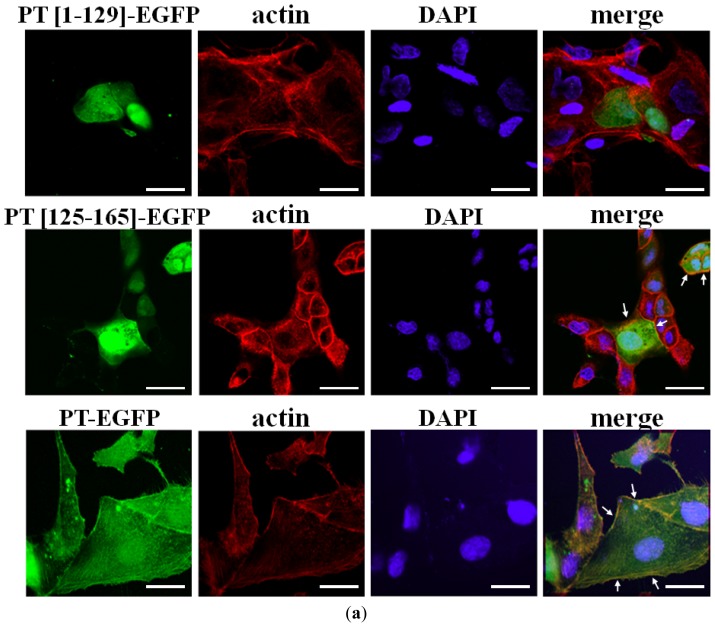

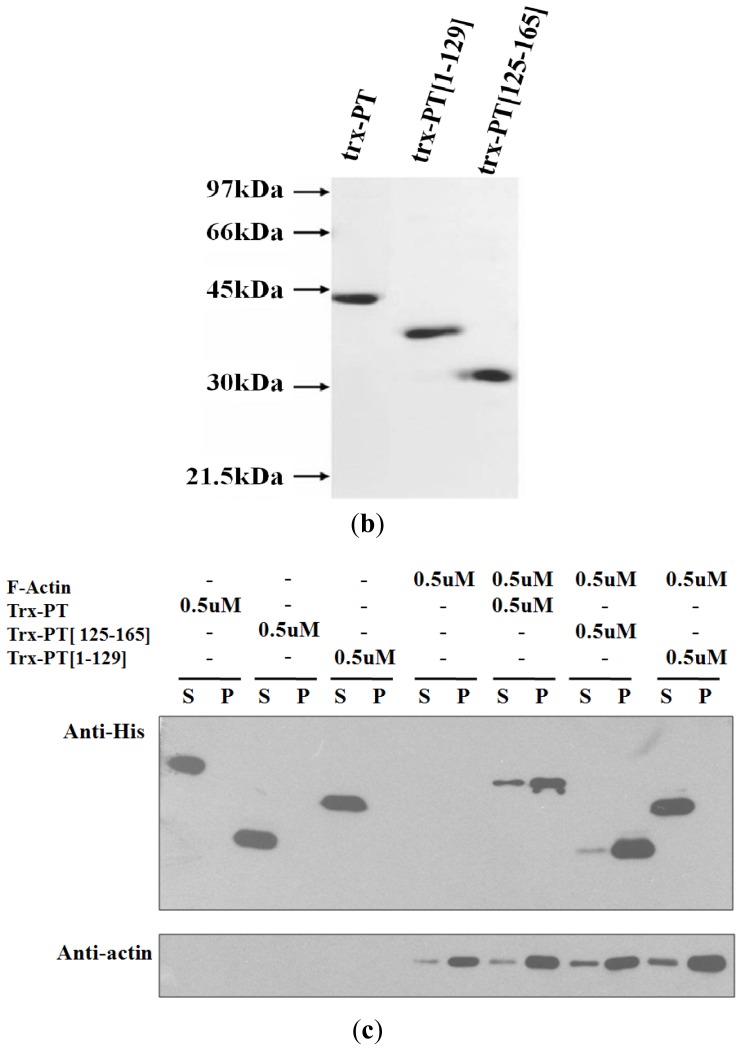
Phostensin (125–165) binds to F-actin. (**a**) Colocalization of phostensin (125–165) and F-actin in MDCK cells. F-actin was stained with rhodamine-phalloidin. Nuclei were stained with DAPI. Fluorescence for PT-EGFP (green) (or PT(1–129)-EGFP (green) or PT(125–165)-EGFP (green)), F-actin (red), and nuclei (blue) are shown on the merged images. Phostensin or PT(125–165) is conspicuously colocalized with F-actin in the cell periphery, as indicated by the arrow. The bar represents 20 μm; (**b**) SDS-PAGE (12.5%) analyses of trx-PT, trx-PT(1–129) and trx-PT(125–165). Molecular weight markers from top to bottom are phosphorylase b (97 kDa), bovine serum albumin (66 kDa), ovalbumin (45 kDa), carbonic anhydrase (30 kDa) and trypsin inhibitor (21.5 kDa); (**c**) Trx-PT(125–165) co-sediments with F-actin at high speed centrifugal forces (150,000*g*). Protein distributed in the pellet (p) and supernatant (s) were analyzed by SDS-PAGE (12.5%). Trx-PT and trx-PT(125–165) were analyzed by western blotting with anti-(His)_6_ antibodies. To analyze the distribution of actin in the supernatant and pellet, the anti-(His)_6_ antibodies on the blotted membrane were deleted by the striping buffer (0.5 M acetic acid plus 0.5 M NaCl), and the membrane was re-blotted with anti-actin polyclonal antibodies.

The binding of residues 125–155 of phostensin to F-actin was determined by cellular colocalization in MDCK cells transiently expressing PT-EGFP, PT(1–129)-EGFP or PT(125–165)-EGFP. F-actin was stained with rhodamine-conjugated phalloidin (Figure 2a). Both PT-EGFP and PT(125–165)-EGFP were distributed at the peripheral cell junctions and cytoplasm, but colocalized with actin-staining structures at the cell periphery (shown in yellow, Figure 2a). However, PT(1–129)-EGFP, which lacks the actin-binding motif, was widely distributed throughout the cytoplasm and did not colocalize with F-actin at the cell periphery. The results suggest that residues 125–165 of phostensin bind to F-actin.

F-actin can be precipitated at high speed sedimentation (150,000× *g*). We applied co-sedimentation assay to examine whether phostensin binds to F-actin. The co-sedimentation assay was performed on recombinant trx-PT, trx-PT(1–129) and trx-PT(125–165). Recombinant trx-PT, trx-PT(1–129) and trx-PT(125–165) were expressed and then purified to 90% homogeneity, as determined by Coomassie Brilliant Blue staining after SDS-PAGE (Figure 2b). In the absence of F-actin, trx-PT, trx-PT(125–165) and trx-PT(1–129) were present in the supernatant at high speed sedimentation (150,000× *g*) (Figure 2c). In the presence of F-actin, it was observed that most of the trx-PT proteins co-precipitated with F-actin in the pellet. Similarly, most of the trx-PT(125–165) proteins were also co-precipitated with F-actin and present in the pellet. However, in the presence of F-actin, trx-PT(1–129) remained in the supernatant. The trx-tag cannot bind to F-actin [6]. These results suggested that phostensin interacts with F-actin through its *C*-terminal domain, spanning from residue 125 to 165 (Figure 2c). In addition, trx-PT did not change the distribution of actin between the supernatant and pellet at high speed sedimentation (Figure 2c), suggesting that it cannot bind to G-actin.

### 2.3. Single Filament Binding Assay

**Figure 3:**
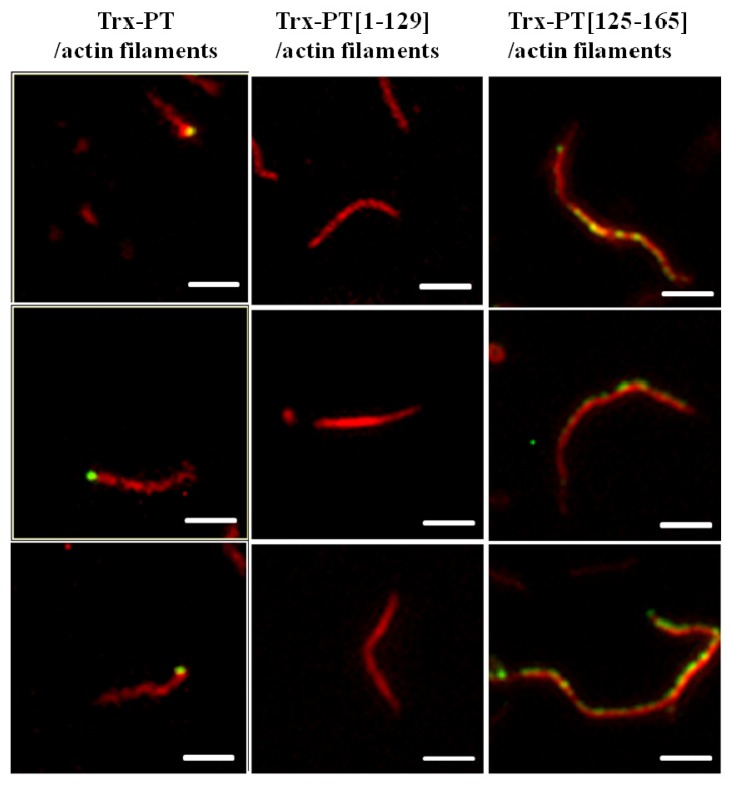
Single filament binding assay. Trx-PT, trx-PT(1–129) or trx-PT(125–165) was conjugated with Alexa-488, which emitted green fluorescence after excitation. Actin filaments were labeled with rhodamine-phalloidin, which emitted red fluorescence after excitation. Alexa-488-labeled trx-PT(1–129) does not bind to actin filaments. Alexa-488-labeled trx-PT(125–165) binds to the sides of actin filaments. Alexa-488-labeled trx-PT binds to the end of actin filaments. Under assay conditions, 96% of Alexa-488-labeled trx-PT were localized at the filament end (*n* = 52); 98% of Alexa-488-labeled trx-PT(125–165) were bound to the sides of actin filaments (*n* = 92). The bar represents 3 μm.

The binding of trx-PT, trx-PT(1–129) or trx-PT(125–165) to F-actin was also examined using a single filament binding assay. The purified proteins were conjugated with Alexa-488 to emit green fluorescence after excitation. Actin filaments were stained with rhodamine-conjugated phalloidin. When Alexa-488-labeled trx-PT was incubated with actin filaments in the presence of rhodamine-conjugated phalloidin, green fluorescence was observed at one end of the actin filaments (Figure 3), similar to the results of a previous study [6]. Colocalization of actin filaments to Alexa-488-labeled trx-PT(1–129) was not observed (Figure 3). However, when Alexa-488-labeled trx-PT(125–165) was incubated with actin filaments in the presence of rhodamine-conjugated phalloidin, some green fluorescence from the sides of the red fluorescent actin filaments was observed. These results suggest that residues 125–165 of phostensin associate with the actin filaments by side-binding, and that one single actin filament can be multiply bound by Alexa-488-labeled trx-PT(125–165) (Figure 3).

**Figure 4:**
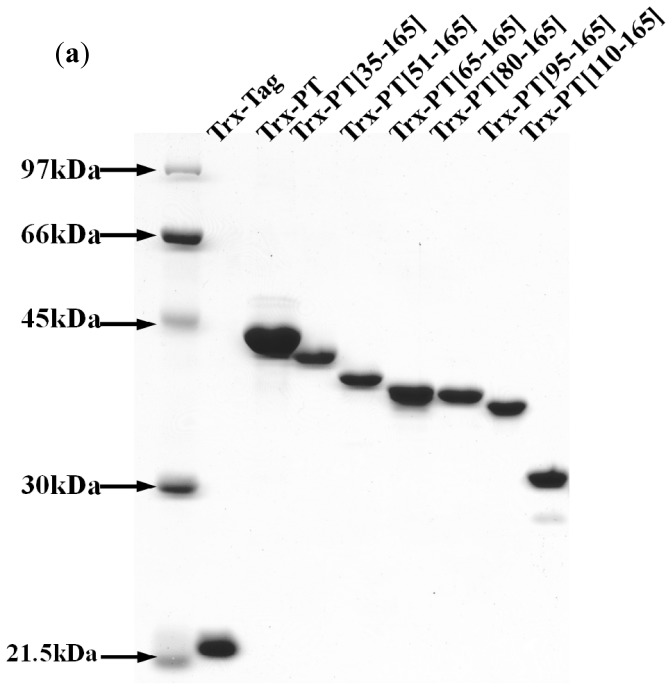

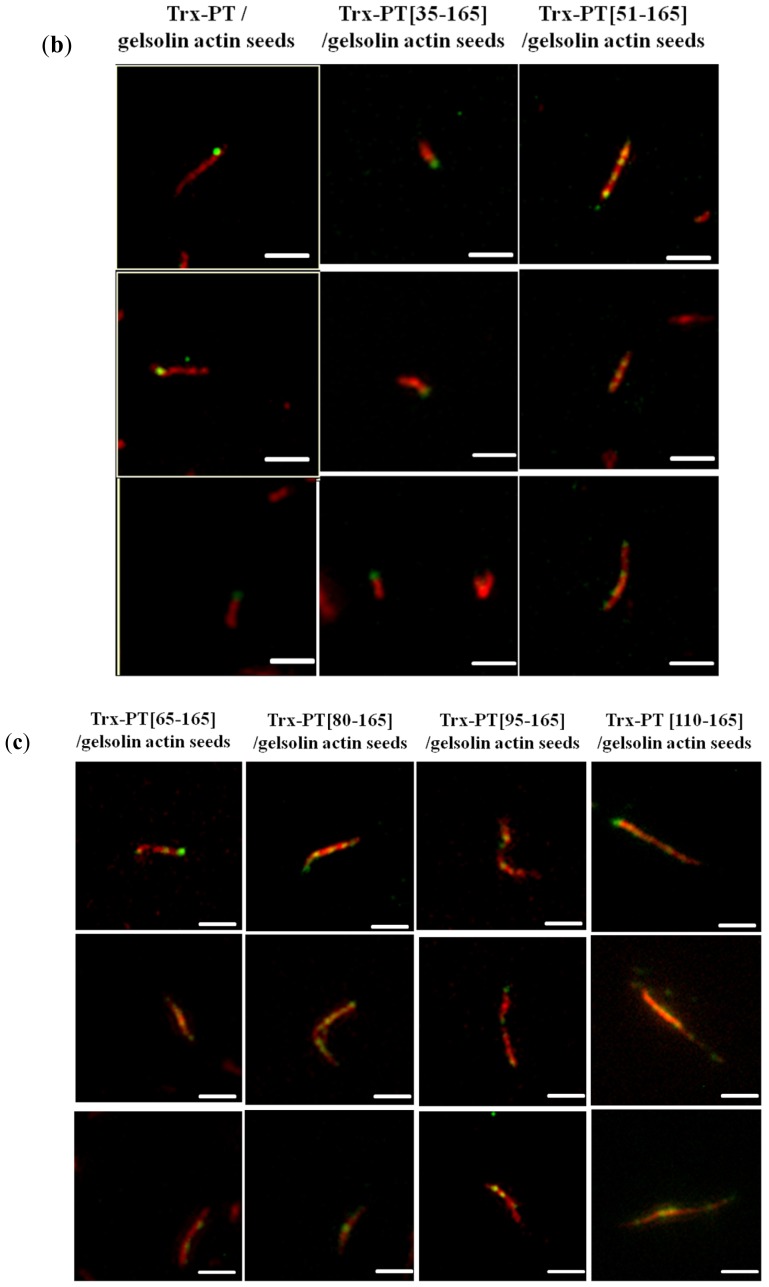
(**a**) SDS-PAGE (12.5%) analysis of phostensin and phostensin mutants, trx-PT(35–165), trx-PT(51–165), trx-PT(65–165), trx-PT(80–165), trx-PT(95–165) and trx-PT(110–165); (**b**) Single filament binding assay. Trx-PT, trx-PT(35–165), trx-PT(51–165), trx-PT(65–165), trx-PT(80–165), trx-PT(95–165) or trx-PT(110–165) was conjugated with Alexa-488 to emit green fluorescence after excitation. The gelsolin-capped actin filaments were labeled with rhodamine-phalloidin to emit red fluorescence after excitation. Trx-PT(51–165), trx-PT(65–165), trx-PT(80–165), trx-PT(95–165) or trx-PT(110–165) binds to the sides of gelsolin-capped actin filaments, while trx-PT or trx-PT(35–165) binds to the ends of the gelsolin-capped actin filaments. Under assay conditions, 96% of Alexa-488-labeled trx-PT(35–165) were localized at a filament end (*n* = 55); approximate 93% to 97% of the other Alexa-488-labeled phostensin mutants were bound to the actin filaments (*n* = 50–104). The bar represents 3 μm.

Intact phostensin has been reported to cap the pointed ends of actin filaments [6]. Unlike intact phostensin, which caps the pointed ends of F-actin, the *C*-terminal region of phostensin, containing the actin-binding motif, can multiply bind to the sides of actin filaments, suggesting that other region(s) of phostensin may play a critical role in capping the pointed ends of actin filaments. To test this hypothesis, we prepared *N*-terminal sequence-deleted phostensin mutants, including trx-PT(35–165), trx-PT(51–165), trx-PT(65–165), trx-PT(80–165), trx-PT(95–165) and trx-PT(110–165). SDS-PAGE analysis showed that the recombinant proteins were at least 90% pure (Figure 4a). The gelsolin-capped actin filaments were incubated with each of the Alexa-488-conjugated phostensin mutants. Green fluorescence was observed at the end of the actin filaments associated with trx-PT and trx-PT(35–165), suggesting that they can both bind to the pointed ends of gelsolin-capped actin filaments (Figure 4b). However, green fluorescence was also observed on the sides of actin filament associated with the trx-PT(51–165) mutant, suggesting that it binds to the sides of gelsolin-capped actin filaments (Figure 4b). Similar results were also observed for trx-PT(65–165), trx-PT(80–165), trx-PT(95–165) and trx-PT(110–165) (Figure 4b), suggesting that residues 35–51 of phostensin play a necessary role in capping of the protein on the pointed ends of actin filaments.

### 2.4. Single Filament Elongation Assay

**Figure 5:**
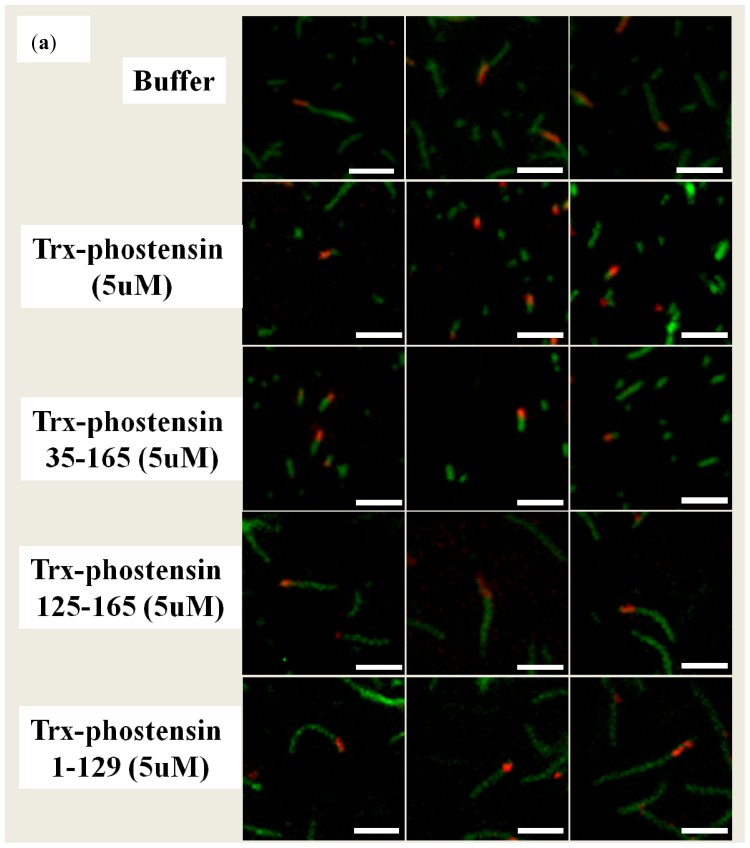

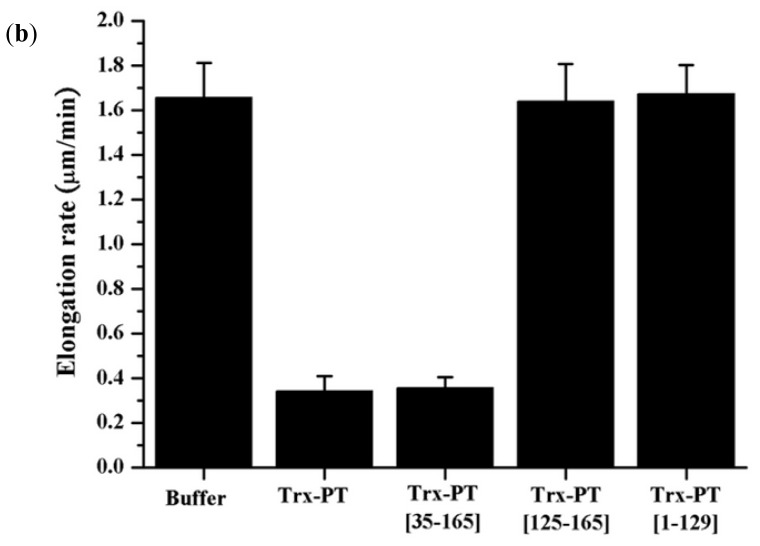
Trx-PT(125–165) and trx-PT(1–129) cannot impede the elongation rates of pointed ends. (**a**) Trx-PT(125–165) and trx-PT(1–129) cannot retard the pointed end elongation of gelsolin-actin seeds. However, under the same conditions, trx-PT and trx-PT(35–165) impede the elongation rates of pointed ends. The bar represents 3 μM; (**b**) The elongation rates (mean ± SD) of pointed ends in the presence of buffer (1.66 ± 0.16 μm/min, *n* = 50), trx-PT in buffer (0.34 ± 0.07 μm/min, *n* = 60), trx-PT(35–165) in buffer (0.36 ± 0.05 μm/min, *n* = 69), trx-PT(125–165) in buffer (1.64 ± 0.17 μm/min, *n* = 65) or trx-PT(1–129) in buffer (1.67 ± 0.13 μm/min, *n* = 50). The lengths of the growing actin filaments (green fluorescence) were obtained from data presented in (a).

If trx-PT(125–165) associates with F-actin by side-binding and trx-PT(1–129) does not bind to F-actin, it is reasonable to infer that both proteins will not retard elongation of pointed ends in F-actin. To confirm this inference, we performed single filament elongation assay on trx-PT(125–165) and trx-PT(1–129). Gelsolin-actin seeds were pre-associated with rhodamine-phalloidon and showed the red fluorescent filaments. The elongation reaction was initiated at the pointed ends of the red actin filaments in the presence of G-actin and Alexa-488-phalloidin. The newly polymerized actin filaments from the pointed ends of the red gelosin-actin seeds were bound with Alexa-488-phalloidin, showing green fluorescent filaments (Figure 5a). The lengths of green fluorescent filaments were divided by the lapsed time for elongation rates. In the absence and presence of trx-PT, the elongation rates of pointed ends in gelsolin-actin seeds were 1.66 ± 0.16 (*n* = 50) and 0.34 ± 0.07 (*n* = 60) μm/min, respectively (Figure 5b). Trx itself did not affect the elongation rates of pointed ends [6]. In the presence of trx-PT(35–165), the pointed end elongation rates were reduced to 0.36 ± 0.05 (*n* = 69) μm/min (Figure 5a,b). This result demonstrated that trx-PT(35–165) can block the pointed ends and retard the elongation from pointed ends. However, in the presence of trx-PT(125–165) and trx-PT(1–129), the elongation rates of pointed ends in gelsolin-actin seeds were 1.64 ± 0.17 (*n* = 65) and 1.67 ± 0.13 (*n* = 50) μm/min, respectively (Figure 5a,b). Apparently, both trx-PT(125–165) and trx-PT(1–129) did not affect the elongation of pointed ends. Trx-PT(1–129) did not bind to the pointed ends, lacking the ability to retard the pointed end elongation. Trx-PT(125–165) associates with the F-actin by side-binding, but it cannot retard the elongation of pointed ends.

The actin-binding motif is present in G-actin binding proteins, F-actin barbed end-capping proteins and F-actin severing proteins [10]. However, it is not known whether this motif is also present in the F-actin pointed capping protein. In this study, we determined that phostensin, an F-actin pointed end-capping protein, also contains an actin-binding motif. The molecular basis for the interaction between the actin-binding motif and G-actin has been elucidated from results obtained from the crystallographic structure of the gelsolin segment 1-actin complex [14]. Gelsolin, an F-actin severing and barbed end-capping protein, contains six independently folded segments [15]. Segments 1 and 4 each contain an actin-binding motif (Figure 1). The segment 1 fragment of gelsolin binds to monomeric actin, with two Ca^2+^ ions trapped in the complex. Ca^2+^ is bound to both actin and the actin-binding motif of segment 1 within a low-affinity site. The carboxylate side chains of Asp-109, located in the actin-binding motif, and Glu-167 of actin interact with Ca^2+^. In the higher-affinity site, Ca^2+^ is bound within segment 1, (*i.e*., residues 35, 65, 66, 97 and 145) [14,15]. Unlike segment 1, phostensin binds to F-actin in a Ca^2+^-independent manner (data not shown). Sequence alignment suggests that Ca^2+^ cannot be trapped in a phostensin-actin complex. In phostensin, Asp-109 of the actin-binding motif in segment 1 is replaced by Gln-149 (Figure 1). Replacement of Asp-109 by Gln completely disrupted the ligation of the intermolecular calcium ion, forming a bridge between G-actin and segment 1 [16]. The higher-affinity Ca^2+^-binding residues of segment 1 are not completely homologous to those found in phostensin.

G-actin folds into four subdomains [17,18]. The actin-binding motif of segment 1 folds into a structure with a β-strand-loop-long-α-helix conformation. The sequence surrounding Ile-103 forms an apolar patch that specifically binds to a cleft formed at the interface between actin subdomains 1 and 3 [14]. The branched hydrophobic side chain of Ile-103 inserts into the hydrophobic environment formed by actin subdomains 1 and 3. A key residue, that is, Ile-103 of segment 1, is replaced by Leu-143 in phostensin (Figure 1). The other key residues of the actin-binding motif between segment 1 and phostensin are highly conserved (Figure 1). An atomic model of segment 1, complexed with actin filaments, has been deduced from X-ray diffraction data [14,18], suggesting that the cleft binding to the actin-binding motif of segment 1 remains present on the surface of the fiber after G-actin polymerizes into F-actin [14]. This possibility could explain our finding that only the *C*-terminal region of phostensin, which contains the actin-binding motif, multiply binds to the sides of actin filaments (Figure 3). Each actin unit of the filament may contain one binding site for the consensus actin-binding motif.

Thymosin β4 contains 43 amino acids with the molecular weight of approximate 5 kDa. Thymosinβ4 is a monomeric actin-binding protein. Several lines of evidence have indicated that thymosin β4 exhibits an unfolded structure in solution. Upon binding to G-actin, thymosin β4 is induced to fold into two α-helices, located at each terminal end of the protein, linked by an unfolded conserved motif, ^17^LKKTET^22^. The *N*-terminal helix with the conserved motif and the *C*-terminal helix have been proven to bind to the barbed and pointed ends of G-actin, respectively [19,20]. The pointed end-binding region of thymosin β4 has been assigned to the sequence, M-(X)_2_-I-(X)_2_-F-X-K-X-KLKK. No homologous sequence like thymosin β4 was found in phostensin. Tropomodulin is an F-actin pointed end-capping [21]. Tropomodulin contains two actin filament pointed end-capping domains. One is the tropomyosin-regulated actin capping that is present in the unstructured region at the *N*-terminal end, ranging from residue 35 to 130. The other actin capping is present at the *C*-terminal end that involves residues between 322 and 359. Although some of the residues between 117 and 132 in the tropomodulin are homologous with the conserved actin-binding motif of phostensin, this region seems to have no significant effect on the actin filament pointed end-capping activity of the *C*-terminal end [21].

## 3. Experimental Section

### 3.1. Materials

Tris, dithiothreitol, ATP, EGTA, MgCl_2_, NiCl_2_, glucose, glucose oxidase, catalase, methylcellulose and imidazole were purchased from Sigma-Aldrich (St. Louis, MO, USA). Blue Sepharose and metal-chelating Sepharose were purchased from Amersham Pharmacia Biotech (Fairfield, CT, USA). Actin, Actin-Binding Protein Biochem Kit BK001 and gelsolin were purchased from Cytoskeleton, Inc. (Denver, CO, USA). The plasmid, phostensin-EGFP (pPT-EGFP), was prepared as described [1]. Rhodamine-phalloidin and Alexa-488-phalloidin were purchased from Invitrogen (Carlsbad, CA, USA).

### 3.2. Fluorescence Microscopy

Phostensin (1–129) (PT(1–129)) and PT(125–165) cDNAs were amplified by polymerase chain reaction (PCR) using the primers provided by the manufacturer. The resulting products were digested with *Eco*RI/*Bam*HI and subcloned into pEGFP-Cl to generate the pPT(1–129)-EGFP and pPT(125–165)-EGFP plasmids, respectively. Madin-Darby canine kidney (MDCK) epithelial cells were transfected with pPT-EGFP, pPT(1–129)-EGFP or pPT(125–165)-EGFP and maintained in Dulbecco’s Modified Eagle’s medium supplemented with 10% fetal calf serum and antibiotics (*i.e*., 25 penicillin at U/mL and 25 U/mL streptomycin) at 37 °C with 5% CO_2_. Cell growth was maintained on slides coated with collagen and blocked with skim milk, following the methods previously described [1]. For staining of polymerized actin, a 1:200 dilution of rhodamine-conjugated phalloidin was used. Cells were analyzed with a Zeiss LSM 510 confocal microscope.

### 3.3. Actin Co-Sedimentation Assay

The Actin-binding Protein Biochem kit BK001 (Cytoskeleton, Inc. Denver, CO, USA) was used to analyze F-actin binding. Briefly, trx-PT (1 μM), trx-PT(125–65) (1 μM) or trx-tag (1 μM) was incubated with F-actin (1 μM), polymerized in a polymerization buffer (5 mM Tris-HCl (pH 7.5), 2 mM CaCl_2_, 0.1M KCl, 1 mM MgCl_2_, and 1 mM ATP) for 30 min at 25 °C, and centrifuged at 150,000× *g* for 1.5 h at 4 °C. Supernatants and pellets were analyzed by western blotting using anti-(His)_6_ or anti-actin antibodies.

### 3.4. Preparation of Recombinant Thioredoxin-Phostensin (trx-PT) and Phostensin Mutant Proteins

Full-length phostensin cDNA was amplified by PCR, and the resulting products were digested with *Nco*I and *Eco*RI and then subcloned into pET32a. For preparation of the recombinant trx-PT, *E. coli* BL21(DE3) was transformed with recombinant pET-32a encoding trx-PT. The transformed bacteria were grown in LB broth with ampicillin (0.1 g/L) and induced with 1 mM isopropyl-β-thiogalactoside for 4 h at 37 °C. Cells were harvested by centrifugation, resuspended in 100 mL of 20 mM Tris-HCl buffer (pH 7.9) containing 0.5 M NaCl, 0.2 mM phenylmethylsulfonyl fluoride, 0.02% NaN_3_, 4 mM benzamidine and 0.5 mM imidazole, and lysed using a French press. Trx-PT was purified from the crude lysate by sequential separation on nickel Sepharose and Mono-Q columns. The purified protein fractions were pooled, concentrated by ultrafiltration and dialyzed against polymerization buffer (5 mM Tris-HCl (pH 7.5), 2 mM CaCl_2_, 0.1 M KCl, 1 mM MgCl_2_ and 1 mM ATP). Phostensin was stored at 4 °C before use. Preparation of the recombinant trx-PT mutant proteins, including trx-PT(1–129), trx-PT(35–165), trx-PT(51–165), trx-PT(65–165), trx-PT(80–165), trx-PT(95–165), trx-PT(110–165) and trx-PT(125–165] followed the method described above. The cDNAs for each phostensin mutant were amplified by PCR. Each resulting product was digested with *Nco*I and *Eco*RI and subcloned into pET32a.

### 3.5. Alexa-488 Conjugation

Before Alexa-488 conjugation, the polymerization buffer was replaced with 500 mM sodium bicarbonate (pH 9.5) using dialysis. One milligram of either trx-PT or mutant protein was dissolved in 1 mL bicarbonate buffer, wrapped in foil and incubated with 80 μg Alexa Fluor 488 tetrafluorophenyl ester (Alexa-488) at room temperature for 2 h. After the incubation, excess reagents were removed by dialysis in polymerization buffer. The labeling efficiency has been estimated. Around 0.21~0.25 molecules per protein molecule were conjugated with Alexa-488 (data not shown)

### 3.6. Single Filament Binding Assay

Gelsolin-actin seeds were prepared as described [6]. Actin filaments, prepared from 1 μM G-actin, were incubated with rhodamine-conjugated phalloidin (1 μM) with the addition of Alexa-488-labeled trx-PT (5 μM) or Alexa-488-labeled trx-PT mutant protein (5 μM) at 22 °C for 30 min. After dilution with the buffer (1:50; *v*/*v*) containing 10 mM imidazole, pH 7.0, 50 mM KCl, 1 mM MgCl_2_, 100 mM DTT, 100 μg/mL glucose oxidase, 3 mg/mL glucose, 20 μg/mL catalase and 0.5% methylcellulose, samples were immediately applied onto poly-l-lysine (1 mg/mL)-coated coverslips for 90 min and observed on a Nikon Eclipse E400 microscope. The images were captured with a Media Cybernetics CCD camera using Image-Pro Plus 5 software. To measure the effect of phostensin or its mutant proteins on pointed end elongation in single filament assay, 500 nM rhodamine-phalloidin-stabilized gelsolin seeds (1:50) in actin polymerization buffer were incubated with trx-PT (5 μM), trx-PT(35–165) (5 μM), trx-PT(125–165) (5 μM) or trx-PT(1–129) (5 μM) for 1 h, followed by incubation with actin (2 μM) plus Alexa-488-labeled phalloidin (2 μM) for elongation reaction. After 2 min, all components were diluted with the buffer (1:50; *v*/*v*) containing 10 mM imidazole, pH 7.0, 50 mM KCl, 1 mM MgCl_2_, 100 mM DTT, 100 μg/mL glucose oxidase, 3 mg/mL glucose, 20 μg/mL catalase and 0.5% methylcellulose, placed on polylysine-coated coverlips and viewed by fluorescence microscopy [6].

## 4. Conclusions

Phostensin lacks the ability to sever actin filaments, and full-length phostensin only binds to F-actin at the pointed ends (Figures 2b and 3). Phostensin consists of 165 amino acids, and the consensus actin-binding motif is located at the *C*-terminal region between residues 129 to 155. The *N*-terminal residues 1 to 129 of phostensin lack interaction sites for actin filaments (Figures 2a,c, 3 and 5a,b), but are essential for phostensin binding to the pointed ends of F-actin (Figure 4b). Thus, no interaction sites for actin filaments were identified in the *N*-terminal region of phostensin. One possible explanation for pointed-end actin binding is that phostensin might fold into a rigid structure, allowing the *N*-terminal region to sterically hinder the actin-binding motif, thus making it impossible to bind to the sides of actin filaments. Only actin units at the pointed ends provide a suitable configuration for phostensin binding.
